# Journal Citation Reports^®^ 2013 tells headache experts that competitive environment has changed

**DOI:** 10.1186/1129-2377-15-55

**Published:** 2014-09-01

**Authors:** Paolo Martelletti

**Affiliations:** 1 Department of Clinical and Molecular Medicine, Sapienza University, Rome, Italy

## JCR sets out 2013 IF

The recent release of Impact Factors 2013 by Journal Citation Reports (JCR) 2013 confirmed the physiological and progressive growth of The Journal of Headache and Pain, untied from geo-political totems or classification duties.

Also this year the growth has been remarkable, over 18% compared to the past year, matching the one of the headache area journal ranked first on the grid line. The Journal of Headache and Pain, in a global editorial scenario, has now upgraded of one position and is second overall between the four journals dedicated to this area of the clinical medicine. Full details are presented as Additional file 1 to this Editorial.

The Open Access strategic option, as cornerstone of a past editorial choice and strictly connected to the introduction of the Article Processing Charge, has proved us to be the right decision helping the Journal to reach these important results [1].

The global trend is evidently towards Open Access and this is out of any doubt, just like the results obtained from every journal that has made this choice at the right time [2]. In fact the National Institute of Health policy stated that their funded papers should be freely accessed since 2008. Another example of this trend is that charitable organizations such as Wellcome Trust included in its funding policy the support to unrestricted access to the published researches in order to reach the widest possible audience.

Circumstances require us to be cautious, but we have to note a general trend of growth of the four area journals and this tells us that, despite the miserable pipeline of headache drugs and the reduction/unification of scientific events on headache, a huge cultural request towards this area still exists and is rising.

## Strategies upholding TJHP boost

The Journal of Headache and Pain will keep witnessing the opening needs to new research groups who are entering the headache editorial world, preferring it to more generalist journals, with a loyal readership composed by a mixed audience not always interested in headache disorders.

An incoming newness is Transparent Peer Review (TPR), as last strategy that will soon be in our hands and before the readers’ eyes: the reviewers’ reports will be fully readable alongside with the published papers. In times of editorial scandals, just to cite the one that brought disgrace on 66 scientific journals [3], TPR will enforce our position of inclusive attitude, far from any kind of lobbying. In other words, Transparent Peer Review will make TJHP totally transparent, friendly and approachable, keeping it a shop of fine glassware and not baubles.

## Next stop June 2015

If every actor of this success, Authors, Reviewers, affiliated scientific societies – European Headache Federation and Lifting The Burden, my multi-localized Editorial Office and obviously Springer Publisher will keep working with the same enthusiasm and passion as ever, of which I am certain, we will be able to meet again at the next stop and share the joy of being helpful to the headache scientific community.

## Coda

At the end of this year the Editorial Boards will expire and then will be refreshed on the basis of the proved scientific contribution. All headache scientists willing to be on-board for the next term are invited to offer their expertise.

## Supplementary Material

Additional file 1 The Journal of Headache and Pain - 2013. Click here for file
