# Supplementary material for: Journal Citation Reports® 2013 tells headache experts that competitive environment has changed
Source: J Headache Pain. 2014 Sep 1;15(1):55. doi: 10.1186/1129-2377-15-55 (PMC4158336; doi:10.1186/1129-2377-15-55)
Supplement: Additional file 1 — The Journal of Headache and Pain - 2013. [file 1129-2377-15-55-S1.doc]

**Electronic Supplementary Material**

| ***The Journal of Headache and Pain - 2013*** | | | | | |
| --- | --- | --- | --- | --- | --- |
| IF | Immediacy Index | Cited Half-life | Total Cites | Eigenfactor® Metrics | |
| 3.281 | 0.941 | 3.4 | 1492 | Eigenfactor® Score  0.00417 | Article Influence® Score  0.656 |
| **Subject Category *Neurosciences*** | | | | | |
| Median IF | Aggregate Immediacy Index | Aggregated Cited-Half-Life | JHP Ranking | Quartile | |
| 2.918 | 0.864 | 7.7 | 107/251 | Q2 | |
| **Subject Category *Clinical Neurology*** | | | | | |
| Median IF | Aggregate Immediacy Index | Aggregated Cited-Half-Life | JHP Ranking | Quartile | |
| 2.182 | 0.669 | 7.3 | 49/194 | Q2 | |
|  | | | | | |
| **ΔIF JHP vs Median IF Neurosciences = +0.363 and vs Median IF Clinical Neurology = +1.099** | | | | | |
| Source: Journal Citation Reports (2013 JCR Science Edition) <http://admin-apps.isiknowledge.com/JCR/JCR>  Accessed, July 31st, 2014 | | | | | |
